# Giving Patients Choices During Involuntary Admission: A New Intervention

**DOI:** 10.3389/fpsyt.2019.00433

**Published:** 2019-07-04

**Authors:** Erin Burn, Maev Conneely, Monica Leverton, Domenico Giacco

**Affiliations:** ^1^Unit for Social and Community Psychiatry (WHO Collaborating Centre for Mental Health Service Development), Queen Mary University of London, London, United Kingdom; ^2^Division of Psychiatry, University College London, London, United Kingdom

**Keywords:** involuntary treatment, shared decision making, intervention, involuntary patients, reducing coercion, feasibility study

## Abstract

**Background:** People who receive involuntary treatment are some of the most vulnerable in psychiatric services. They are more likely to have poorer social and clinical outcomes and to be disillusioned with and disengaged from care. Research indicates that patients’ experience in the first week of involuntary treatment is a critical period: a better experience of care in the first week predicts better quality of life and reduced readmission 1 year later. Patients have identified involvement in clinical decisions as key to improving their experience of care. The aim of this study was to test the feasibility and acceptability of an intervention to facilitate involvement in decision making for involuntary inpatients called OPeNS (Options, Preferences, Negotiate, and Summarise).

**Methods:** This was a mixed method study. The OPeNS intervention was developed based on previous research carried out by a multidisciplinary team. Clinicians were trained to deliver it to involuntary inpatients. Feasibility indices (rates of participation in the intervention and time required to deliver it) were collected. Patients (*N* = 14) and clinicians (*N* = 5) provided qualitative data on their experience of the intervention in semi-structured interviews which were analysed using thematic analysis.

**Results:** The OPeNS intervention was found to be acceptable by both patients and clinicians and feasible to conduct within the first week of involuntary treatment. Patients’ and clinicians’ experiences of the intervention fall into two themes: ‘Enabling a different dynamic’ and ‘Clashing with usual practices and priorities’.

**Conclusion:** The OPeNS intervention provides a structure that can be used by clinicians across disciplines to facilitate involving involuntary patients in decision making. Although challenges related to changing usual practices were identified, the intervention was received positively and was feasible to conduct in the first week of involuntary treatment.

## Introduction

The number of people detained against their will has been increasing over the past 20 years in many countries (1–5). In 2015–2016 in England, there was a 9% rise from the year before in involuntary admissions, adding up to a total of 63,622 (6). Across the country more than half of all admissions to psychiatric hospitals are involuntary, and in some areas the rates of involuntary admission are as high as 67% (7). Although it is widely practiced, involuntary hospital treatment offers ethical challenges. Deontological and ethical standards allow for involuntary treatment only if it is in the interest of the patient and of the highest quality (8, 9).

Involving patients in decisions about their treatment has been widely advocated as being a core component of good quality care, and it is the third quality statement in the National Institute for Health and Care Excellence (NICE) guidelines (10–13). Shared decision making principles include the sharing of information to reduce power asymmetry between care providers and patients, and to empower patients to be more engaged and active in the process of decision making (14, 15).

Shared decision making is widely advocated and yet concerns have been raised that a significant number of patients are not involved in decisions, in particular patients who are being treated under the Mental Health Act. For example, in England, patients receiving treatment in the community, who had agreed their care with an National Health Service (NHS) clinician, were asked if they were involved in the decision making process. In 2018, only 53% of patients said they were ‘definitely’ as involved in the decision making process as they wanted to be, which was a statistically significant decrease compared to the previous year (16). The figures for involuntary patients’ involvement in decision making are poorer still: in 2015–2016, the Care Quality Commission (CQC) reported that only 29% of involuntary patients felt involved in decisions about their treatment. This suggests that there is a need for interventions to improve the implementation in practice of shared decision making (SDM) principles, and a particular need for interventions to facilitate involving involuntary patients in decisions about their care (17, 18).

Based on the principles of shared decision making, improving patients’ involvement in decision making has been the goal of several interventions developed in psychiatry (10, 19). These interventions have been carried out in depression (20, 21), schizophrenia (22–24), bipolar disorders (25) and across inpatient and community treatment settings (10, 26–28). A Cochrane review identified two randomised controlled trial (RCT) testing interventions to improve shared decision making (29), showing mixed results of clinical efficacy. Loh and colleagues reported that depressed patients in the SDM intervention group receiving treatment in primary care showed increased patient satisfaction; however, this finding was not replicated by Hamman and colleagues in inpatients with schizophrenia (24, 30). Additionally, both studies found no evidence of an effect on either clinical outcomes or readmission rates. More recently in a systematic review and meta-analysis of SDM interventions for patients with psychosis, Stovell and colleagues found improvement in patient empowerment and some more limited support for SDM having an impact on compulsory readmission (23).

These studies indicate that it is possible to improve some outcomes through increasing how involved patients are in decision making. Although some of the studies’ samples included involuntary patients, the interventions were not developed specifically for this population. The structural power imbalance between care providers and patients makes the sharing of all healthcare decision making challenging; however, there are particular additional challenges in the context of involuntary inpatient treatment where many choices have been taken away from patients. The barriers to implementing SDM in involuntary inpatient settings may be substantially different to the barriers in outpatient settings and with voluntary inpatients (31, 32).

This study aimed to test the feasibility of an intervention developed specifically for involuntary inpatients: the OPeNS (Options, Preferences, Negotiate, and Summarise) intervention. Based on the evidence that patients’ experience of the first week of involuntary treatment is a critical period, the OPeNS intervention focused on operationalising the tenets of SDM with a straightforward, structured approach, to be carried out within the first week of involuntary treatment (33, 34).

We aimed to answer the following research questions:

1. How many patients are able to participate in the OPeNS intervention within 1 week of admission?
2. How do patients and clinicians experience the OPeNS intervention session?

## Materials and Methods

### The OPeNS Intervention

The intervention comprises of a 30–60-min meeting between a clinician and a patient that begins in the first week of involuntary treatment and is intended to be offered as part of routine care. In this meeting, a four-step structure that gives the intervention its name is followed. The steps are as follows:

- Give an overview of *Options*: Although patients may be in hospital against their will and there are certain aspects of their care they now legally do not have control of, there are some aspects of life on the ward where they do have options. Patients are given an overview of areas of hospital life and treatment that might be important to them and that they might want to discuss. This included leave from the ward, contact with family, food on the ward and medication (see **Supplementary Appendix A** for the document used). The list serves as a prompt and patients are encouraged to consider anything else that might be important to them. Once the patient has indicated what is most important to them (usually up to three items on the list), the different options within these areas are presented.
- Explore their *Preferences*: In this step, the clinician checks that they understand what the patient’s preferences are, the reasons behind the preferences and what their concerns are.
- *Negotiate*: In this step, the clinician should explain their perspective. In the case of disagreement with the patient’s wishes, the clinician should allow the disagreement to take place, and the patient’s views and concerns about each option to be acknowledged. Both the patient and the clinician are given the space to explain their perspectives, and negotiate an option that is acceptable to both. In the instance that an agreement is not possible, a time should be set to revisit the decisions.
- *Summarise*: Patient and clinician summarise the decision made in the action plan. The action plan is a tangible document: a piece of paper which lists the actions that have been agreed upon in the session (usually up to three actions) as well as the person who is to carry out the action. If they decide to have another session, the date of the next session is recorded. The action planning document is co-produced by the patient and the clinician; it is written by the patient or, if that is not possible, written by the clinician and dictated by the patient.

### Development and Training

The intervention was developed based on previous research including a systematic review (35) and a focus group study (36) by a multi-disciplinary research team. The team included psychiatrists, psychologists, nurses, a lawyer with expertise of the Mental Health Act (MHA), public health experts, research methodologists, patients and carers who worked in consultation with a specifically constituted Lived Experience Advisory Panel (LEAP), which included three patients and two carers who had experience receiving or supporting someone who had received involuntary treatment in the previous year. The intervention training took 3 h and was delivered by DG and MC.

### Design

This was a mixed-method study. Quantitative measures were used to assess the feasibility of the intervention and experience was explored in semi-structured interviews.

*Quantitative.* The main indices of feasibility of the intervention used were the rates of participation in the intervention, the time required to deliver it and the number of items discussed. Eligible patients were approached to take part in the intervention in the first days after their admission in line with the purposive sampling criteria (see the section Context and Sampling for more on sampling). Clinicians recorded the time it took (in minutes) and reported whether there were any disagreements in the session (rated dichotomously as either yes or no). Patients reported their satisfaction with their action plan using a single-item statement ‘my action plan is right for me’, rated on a five-point Likert scale ranging from ‘strongly disagree’ to ‘strongly agree’. Patient involvement was measured by patients selecting one of the following options: ‘I made the action plan’, ‘We made the action plan together’, ‘My clinician made the action plan’. Satisfaction with treatment was measured using the Client’s Assessment of Treatment Scale (CAT) (37), which is a seven-item scale developed to assess patients’ appraisal of in-patient care and includes the items: ‘Do you believe you are receiving the right treatment/care for you?’, ‘Does your therapist/case manager/key-worker understand you and is he/she engaged in your treatment?’, ‘Are relations with other staff members pleasant for you?’. Responses are marked on a horizontal line between the extremes which are labelled from 0 to 10, from ‘not at all’ to ‘entirely’. The CAT has good internal consistency, is considered meaningful to patients, including involuntary patients, and has good predictive validity independent of symptoms (34, 38, 39).

*Qualitative.* We conducted semi-structured interviews with patients and clinicians to explore their experiences of the intervention and their thoughts on barriers and facilitators to its implementation.

#### Context and Sampling

The study took place at Newham Centre for Mental Health which is a part of East London NHS Foundation Trust. Involuntary inpatients were recruited from the inpatient wards into three purposive categories based on their diagnosis: schizophrenia and related disorders (ICD 10 code: F20–F29), mood disorders (F30–F39), personality disorders (F60–F69), mental and behavioural disorders due to psychoactive substance abuse (F10–F19) and unspecified mental disorders (F99) and whether they had a history of previous admission. Patients were recruited consecutively in order to reach the target number for each category. In certain cases, participants could have fit into different purposive categories as they had more than one diagnosis – in this case the main or most recent diagnosis was used.

Diagnosis was a purposive sampling criterion as we wished to avoid that our findings would be biased by an over-recruitment of a specific group of patients with the same diagnosis. We also sought to recruit at least *n* = 4 patients at first admission, as being in hospital for the first time may also influence experience of care and engagement with interventions (40).

Clinicians were also recruited using purposive sampling to maximise variation: nurses, psychiatrists and clinical psychologists were trained to deliver the intervention.

Patients were eligible if it was their first week of involuntary admission under the MHA, fit into one of the purposive categories, were over the age of 18, had the capacity to give informed consent for research and had a sufficient command of the English language to take part in the intervention and in the semi-structured interview.

#### Procedures

Semi-structured one-to-one interviews were used to explore both clinicians’ and patients’ experience of the intervention and to highlight any potential barriers to delivery. A topic guide which was developed with input from the Lived Experience Advisory Panel was used for the interviews, which can be found in **Supplementary Appendix B**. Interviews were conducted by a member of the research team (MC) in a quiet room on the wards at Newham Centre for Mental Health.

The interviews were audio-recorded and transcribed by an external transcription company. Any identifiable information was removed. Transcripts of clinicians’ and patients’ interviews were analysed together using phenomenologically informed thematic analysis (41) to explore their experiences of the interventions and their thoughts on implementing the intervention. A subset of the transcripts was coded inductively by two researchers (MC and ML) for comparison. After comparing and discussing the codes, a coding framework was developed and used to code the remaining transcripts (MC). Themes were created based on the final codes and refined through discussions between authors. Although the transcripts were analysed together, the representation of clinicians and patients in the final themes was examined, and where a theme or sub-themes were particularly frequent or relevant to one group, this was noted.

### Ethical Approval

This study was approved by the London Bridge Research Ethics Committee (Ref: 16/LO/0384).

## Results

**Figure 1:**
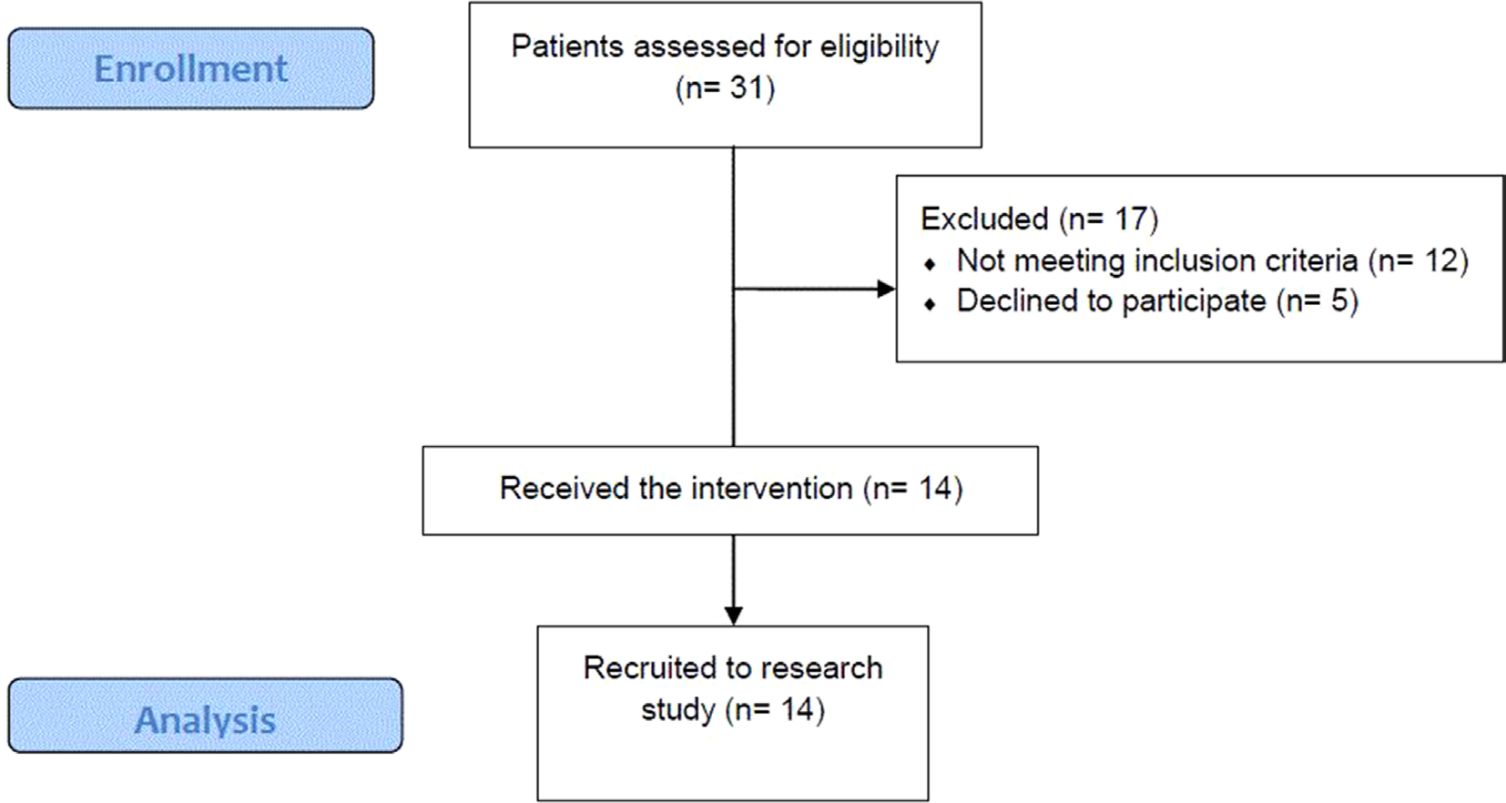
Consort Flow Diagram.

Fourteen of the 19 eligible patients (73.6%) took part in the intervention session. Thirty-one patients were approached, 19 of whom met the eligibility criteria. The most frequent reason for exclusion was patients not speaking sufficiently fluent English (*n* = 7) and lacking the capacity to consent to the research (*n* = 5). Five eligible patients declined to take part in the intervention (see **Figure 1** for consort diagram of patient recruitment). Patients’ average satisfaction with treatment measured using the CAT was 5.45 (on a scale from 0 to 1). The clinicians (*n* = 5) who were recruited to deliver the intervention were also interviewed about their experience. Sociodemographic and clinical details of the patients and clinicians can be found in **Table 1**.

**Table 1:** Sociodemographic and clinical characteristics of participants.

Patients (*n* = 14)	*n* (%) or mean (SD)
Age in years	34.7(0.5)
Gender (% female) Country of birth UK (% UK) 1st Involuntary Admission Length of admission in days	10 (71.4%) 7 (50%) 5 (35.7%) 29.1 (16.8)
*Mental Health Act*	
Section 2	12 (85.7%)
Section 3	2 (14.3%)
*Diagnosis*	
Psychotic disorder	5 (35.7%)
Mood disorder	4 (28.6%)
Personality disorder Substance abuse disorders Unspecified mental disorder	2 (14.3%) 2 (14.3%) 1 (7.1%)
**Clinicians (** ***n*** ** = 5)**	***n*** **(%) or mean (SD**)
Age in years	39.2 (15.1)
Gender (% female)	3 (60%)
Years working in inpatient care Years working in mental health	8.9 (12.6) 14.9 (12.9)

### Intervention Characteristics

The average length of each intervention session, as recorded by clinicians, was 29.6 min. Following the intervention, 10 patients reported that they made their action plan in collaboration with the clinician and 4 stated that the clinician made their action plan. Sixty-four percent of patients reported that they either agreed or strongly agreed that the action plan was right for them (see **Table 2**).

**Table 2:** Intervention characteristics.

Intervention session	
**Mean length of session (min)**	29.6 min
**Carer present**	2 (14.3%)
**How many items discussed** One Two Three Four Nine Not recorded	*n* = 1 (7.1%) *n* = 5 (35.7%) *n* = 4 (28.6%) *n* = 1 (7.1%) *n* = 2 (14.3%) *n* = 1 (7.1%)
**Were there any disagreements between clinician and patient?** Yes No Missing	*n* = 4 (28.6%) *n* = 9 (64.3%) *n* = 1 (7.1%)
**Patient involvement** I made the action plan We made the action plan together My clinician made the action plan	*n* = 0 *n* = 10 (71.4%) *n* = 4 (28.6%)
**Patient satisfaction – my action plan is right for me** Strongly disagree Disagree Neither agree nor disagree Agree Strongly agree	*n* = 1 (7.1%) *n* = 1 (7.1%) *n* = 3 (21.4%) *n* = 6 (42.9%) *n* = 3 (21.4%)

### Qualitative

Patients’ and clinicians’ experiences of the OPeNS intervention are summarised in two themes: ‘enabling a different dynamic’ and ‘clashing with usual practice and priorities’ (see **Table 3**).

**Table 3:** Themes and subthemes.

Theme	Subtheme
Enabling a different dynamic	Having a voice A space to talk about something different Making action-focused and patient-led decisions
Clashing with usual practices and priorities	Competing priorities on the ward The earlier the better?

## Enabling a Different Dynamic

### Having a Voice

Having a space to talk was appreciated by both clinicians and patients, and the structure of the OPeNS intervention, which begins with both clinician and patient considering the same list of options (**Supplementary Appendix A**), was seen by both groups as giving some power back to the patient. Being listened to and having their views heard was something underlined as being particularly important to patients.

‘The most important thing is that you’re giving me the opportunity to air my view, my experience’ – Patient 10

‘I think on one basic level, simply having a space to talk was valued by the patients, [ … ] and also really specifically to actually make decisions and actually have a say in what they wanted in their care was really valuable and quite different to what they had experienced before’ – Clinician 06

Some reflected that the patients’ involuntary status may contribute to them not being included in psychosocial interventions, and therefore maybe not having an opportunity to be engaged in conversations about their care.

‘It was quite interesting just getting into their minds, because often when someone is under section they don’t refer to psychology.’ – Clinician 03

As well as having a space to be heard, patients saw the *quality* of listening as important.

‘Like they can listen, because it obviously is their job to listen, but then there is listening to understand, and making sure that what you’re saying happens, you know what I mean?’ – Patient 06

The negotiation phase of the OPeNS intervention was seen as positive, irrespective of whether the patient and the clinician agreed with each other about the actions to take; allowing the space for each party’s view to be heard improved understanding in both directions and improved the therapeutic relationship.

### A Space to Talk About Something Different

The OPeNS intervention was experienced as a new and different type of meeting to other routine meetings on the ward. The main reason it was set apart for patients and clinicians was because a) the topics open for discussion were chosen by the patients and often not what is routinely discussed, and b) because it was action-focused.

‘She gave me a choice of three things, I thought it was good to have that. There’s things that people don’t talk about for whatever reason [ … ] you have to have the room or the borders to facilitate that because sometimes not everyone does’ – Patient 08

‘Overall it is good because the patient can say something about some topics that are interesting to them, that they cannot talk about usually. Most of these topics are not discussed in the ward round, we look at the more clinical point of view’ – Clinician 04

### Making Action-Focused and Patient-Led Decisions

The intervention starting with a choice being made by patients, and not an agenda being set by clinicians, was seen as key to creating a neutral space for a different channel of communication. The physical, printed list of options that were presented to patients at the beginning of the intervention (**Supplementary Appendix A**) was seen as essential to allow the decision about the focus of the discussion to come from the patient.

‘The way it was brought to patients, being like “you can lead this, you can lead the decisions that are made,” within the parameters obviously, I think just us coming like “I’m going to listen to you and you’re going to lead this”, I think that was really empowering, and quite surprising’ – Clinician 06

‘It’s really nice if you know you have these different topics that you can talk about with the doctor, the nurse’ – Patient 01

## Clashing With Usual Practices and Priorities

Despite reporting a positive experience of the intervention, and agreement with the idea of the intervention, patients and clinicians also expressed some concerns regarding how it was carried out in practice and could fit into routine care. These barriers identified were the busy nature of acute inpatient care and staff attitudes to inpatients and their mental state in the first days after their admission.

### Competing Priorities on the Ward

A lack of protected time was consistently stated as a major barrier that needs to be overcome to ensure successful implementation of the intervention. It was common within the patient and clinician’s narratives that the ward can be a busy environment which can affect the time and resources (e.g. a private room) at the clinician’s disposal.

‘In terms of my regular day to day activity, I do work on a busy ward and so it can be difficult to allocate specific time for activities such as the intervention just due to the unpredictable nature of the sessions that I have to do within the day.’ – Clinician 03

‘The staff are too busy [ … ] There’s about five patients per staff. You should have seen yesterday, there’s only two staff to ten patients’ – Patient 12

Barriers were also identified by both patients and clinicians concerning staff motivation to promote and deliver the intervention. As clinicians are the vehicles of change, for the intervention to be effective, the staff need to be engaged and exhibit the right attributes to promote honest discussion.

‘It’s not the environment it’s just the attitude to work’ – Patient 03

‘I see more barriers with the staff. I never have any problem with the patient. For some reason, it seems that nothing can change, it is more a matter of culture in the wide sense of the word.’ – Clinician 04

### The Earlier the Better?

Despite the potential of competing ward priorities, patients saw it as essential that the intervention takes place early, as it is a way of giving a sense of agency back to a patient who has just been brought into hospital against his or her will. It was also seen as a way of setting a positive tone for the admission and helped orient the patient on the ward.

‘I think it should happen like the first probably two to three days on the ward, because at that point it’s like you’re, this is basically home for however long you, you’re going to be here. But the first couple of days are majorly the key, isn’t it?’ – Patient 06

However, several patients and clinicians also felt that not everyone was well enough to engage in an intervention immediately following their involuntary admission – in part because this often involved a change in medication.

‘The reason why I keep going on about the timing is because you could not have done it for me about the day I came in because I slept for what seemed like two days, because of the injection, I’ve never had any horrible injection like that I was just zombie like.’ – Patient 03

‘I think offering time, so maybe a few days to, hopefully, if that is what’s causing or driving the situation in terms of their mental state, maybe offering a few days might give a chance, if they’ve been prescribed medication for example, if that’s going to help with their symptoms then they may be more amenable to discussions later on again.’ – Clinician 03

## Discussion

### Main Findings

This study tested the feasibility of an intervention to facilitate involvement in decision making for involuntary inpatients. Although it is common-place for psychosocial interventions to be left to later on in involuntary admission, this study has demonstrated that a large proportion of involuntary patients were both able and willing to take part in the OPeNS intervention in the first week of involuntary admission. The intervention was positively received by both clinicians and patients, as it allowed a different type of interaction to occur between the patient and the clinician. The timing of the intervention was seen as important, as it set the tone for further treatment during the admission, helped orient the patient to the ward and provided a positive reciprocal relationship between the patient and the clinician from the start. However, there were concerns that the wards’ busy environment, the shortness of staff and clinicians’ attitude might make the intervention difficult to implement. The quantitative measures indicate that it is feasible to involve involuntary patients in decisions about their care from the first days of their admission.

### Strengths and Limitations

This study has several strengths. First, this is the first known study to explore the feasibility of structured shared decision making practices during the first week of involuntary hospitalisation. It is also part of a very small number of interventions which have been developed and tested on involuntary inpatients, although none of them started so early during admission (35). Additionally, the intervention was developed in collaboration with a specifically formed Lived Experience Advisory Panel, consisting of both patients and carers who had recent experience of receiving, or supporting someone who had received involuntary treatment. The views of people who have experienced involuntary treatment were important in each stage of the intervention’s development, including the initial grant application. We, however, would have liked to have had more lived experience involvement, for example in the analysis of the qualitative data and in the training of the clinicians; however, due to limitations in funding and timescales, this was unfortunately not possible.

This study has the following limitations. Firstly, the intervention was tested only in a small sample recruited from one inpatient psychiatric hospital in Newham, East London. The area served by the hospital is multicultural and has a high deprivation index. These factors may have affected involvement in decision making and limit the generalisability of the study (42). Feasibility of the intervention should be tested in other contexts.

Secondly, the number of patients and clinicians involved was limited, so we cannot exclude the possibility that in both groups, selection biases towards a higher openness to shared decision making practices might have influenced the mainly positive experiences.

Thirdly, we only tested one session of the intervention whilst the procedures would lend themselves to follow-up sessions in which actions are reviewed and, if required, amended. Having only tested one session, we are unable to conclude as to whether there may be any factors which might affect whether the decisions are implemented or not.

### Comparison With the Previous Literature

Existing research in outpatient and inpatient settings has demonstrated that SDM interventions are feasible for most patients and that outcomes including empowerment and patient satisfaction with care can be improved (22, 30). However, this is the first study to develop and test an intervention specifically developed for involuntary inpatients.

Previous research has shown that involving involuntary patients in decisions is linked to improved experiences and clinical outcomes. This has been established both through observational (33, 34, 43) and in a small number of experimental studies (35). The involvement of patients in decision making is also supported by policies and is regarded as a component of best clinical practice.

Participants reported that having a single session to improve shared decision making had an important effect on the dynamic of the patient-clinician relationship. This might mean that shared decision making is not only good practice for these patients and at that point of admission, but also could have a transformative effect on patient engagement and on their experience of care.

As would be expected from an intervention reported to have a transformative effect on usual practice, the intervention was reported by clinicians to clash, to some extent, with clinical routines within an inpatient ward. This can explain the lack of implementation of shared decision making with involuntarily hospitalised patients and why patient involvement in decision making is not always implemented in practice. The intervention may thus be seen as a structure to facilitate the implementation of shared decision making.

### Implications

Involving involuntary inpatients in decision making is an ethical requirement. However, this study revealed that when introducing procedures to help implementation, concerns may arise as to whether it fits with the wards’ routines. Hence, introducing and standardising shared decision making practice is likely to be a task that goes beyond simple quality assurance, warranting standardised interventions and, probably, far-reaching changes in work cultures and arrangements within wards.

Despite the identified barriers, efforts to standardise and implement shared decisions making practices during involuntary treatment may pay off by improving patients’ experiences of care and their clinical outcomes. The fact that a single shared decision making session was perceived by clinicians and patients to enable a different dynamic and facilitate an improved therapeutic alliance is highly promising.

The straightforward single-session intervention that we developed has shown to be feasible in the first week of admission. Yet, more needs to be done in order to improve long-term outcomes. Research suggests that patient-led care planning at discharge and appropriate follow-up after discharge are the most effective existing interventions for these patients (35, 44). A comprehensive intervention in which shared decision making in hospital is associated to these two other components may be a way forward to achieve maximum effect.

Another important learning from our study is that it is possible to carry out research with patients within 1 week of involuntary admission. In our design we offered the intervention as standard practice; hence, all eligible patients were offered the intervention, even if not providing consent to research procedures. This methodology has previously been used with patients who are treated for acute medical conditions (45) and may be an important way forward for research in involuntary patients. Indeed, including only patients who are able to consent to research is likely to carry a significant selection bias and findings which are of little interest to improve practice for the large majority of involuntarily admitted patients.

## Ethics Statement

This study was approved by the London Bridge Research Ethics Committee (Ref: 16/LO/0384).

## Author Contributions

EB wrote the first draft, and all of the authors contributed to its revisions. DG led the design of the intervention and was the chief investigator on this study. MC interviewed the patients and clinicians, and MC and ML conducted the thematic analysis.

## Funding

This study was funded by the National Institute for Health Research (NIHR) Programme Development Grants for Applied Research (Ref.: RP-DG-1214-10004). Giacco and Burn were supported by the NIHR Collaboration for Leadership in Applied Health Research and Care North Thames at Barts Health NHS Trust. The views expressed are those of the author(s) and not necessarily those of the NHS, the NIHR or the Department of Health.

## Conflict of Interest Statement

The authors declare that the research was conducted in the absence of any commercial or financial relationships that could be construed as a potential conflict of interest.
