# Supplementary material for: Giving Patients Choices During Involuntary Admission: A New Intervention
Source: Front Psychiatry. 2019 Jul 4;10:433. doi: 10.3389/fpsyt.2019.00433 (PMC6620234; doi:10.3389/fpsyt.2019.00433)
Supplement: Supplementary file 1 [file DataSheet_1.pdf]

Medications

[time of day, type of medication (liquid or tablet), sleeping tablets, painkillers, side effects, depot]

Activities on the ward

[groups on the ward, activity supplies, computer room, member of spiritual team, prayer room]

Personal Belongings

[can request pyjamas or slippers from the hospital, choose what is brought, personal belongings kept in the staff room, use staff phone 7am-10pm, TV]

Leave

[AC: escorted or unescorted 8am-8pm, request to book a taxi, escorted leave activity]

Legal Rights

[request mental health advocate, phone advocate, request advocate, solicitor choice]

Physical Health

[leave to go to the gym (WR), discuss additional checks (WR, WD), specialist (WR)]

Food on the ward

[Choose meal for next day, request to see a dietician (NS)]

Safety

[choose to be escorted on leave, whether room is locked, request to swap room, level of observation (NS, WD, WR)]

Contact with friends/relatives

[visit family and friends 4pm-8pm, invite friend or family to WR, choose what information is shared, family room]

Other
