# Supplementary material for: Giving Patients Choices During Involuntary Admission: A New Intervention
Source: Front Psychiatry. 2019 Jul 4;10:433. doi: 10.3389/fpsyt.2019.00433 (PMC6620234; doi:10.3389/fpsyt.2019.00433)
Supplement: Supplementary file 2 [file DataSheet_2.pdf]

TOPIC GUIDE: AN INTERVENTION TO FACILITATE PATIENT INVOLVEMENT IN DECISION MAKING DURING COERCIVE CARE

PATIENT INTERVIEW

1. INTRODUCTION & SETTING GROUND RULES (5 MINS)

Thank the participant for his/her availability, introduce researchers and explain:

- Nature and focus of research i.e. the interview is about discussing the participant's experience of receiving the intervention that aims to facilitate patient involvement in decision making about their care.
- Confidentiality: The name of the participant will only be known by the researchers in the present interview and not be revealed to anyone
- The session will be audio-recorded, transcribed and analysed by researchers using NVivo software, one of the most used widely software for qualitative analysis
- The participant will be identified by an ID and all potentially identifying information will be removed

GROUND RULES

- You do not have to share any information that you don't want to
- There are no right or wrong answers
- All conversations must remain confidential
- Mobile phones should be off or on silent-vibrate whilst the group is working
- Any questions?
- Start tape recorder

2. BACKGROUND (5 MINS)

Aim: Icebreaker

- Ask the participant to say a brief word about themselves:
    - First Name
    - Whether they have taken part in an interview before

3. DISCUSSING BENEFITS & PROBLEMS OF IMPLEMENTING THE INTERVENTION (UP TO 30 MINS)

Aim: To understand participants' experiences of receiving the intervention, focusing on the benefits and problems of the intervention.

This intervention is aimed to facilitate patient involvement in the decision making process of your care.

- What do you think are the benefits of implementing the intervention session? Did you find the session useful?
- Did you encounter any problems during the intervention session?

  Were there any problems related to the ward environment or type of ward?
- Was your carer present during the intervention session? If so, what do you think were the benefits of including carers in the intervention session? Were there any problems with including the carer in the session?

4. DISCUSSING PRACTICAL ISSUES RELATED TO THE INTERVENTION (UP TO 40 MINS)

Aim: to understand participants' opinions on the practicalities of receiving the first intervention session.

- Why did you agree to take part in this intervention session?
- How did you deal with perceived disagreement with the clinician during the intervention session?
- Was it helpful to receive this intervention session in your first week of being admitted to hospital?
- Would you attend further intervention sessions?

5. CONCLUSION / DEBRIEF (10 MINS)

- Thinking about the conversations we have had today about the plan, which **one thing** do you think is the biggest benefit of the intervention session?
- What do you think is the most important barrier to it?

- Thank participant for his/her contribution
- Remind participant that all information is confidential
- Information on project timescale (analysis of data, preparation of a report)
