# Supplementary material for: Giving Patients Choices During Involuntary Admission: A New Intervention
Source: Front Psychiatry. 2019 Jul 4;10:433. doi: 10.3389/fpsyt.2019.00433 (PMC6620234; doi:10.3389/fpsyt.2019.00433)
Supplement: Supplementary file 3 [file DataSheet_3.pdf]

TOPIC GUIDE: AN INTERVENTION TO FACILITATE PATIENT INVOLVEMENT IN DECISION MAKING DURING COERCIVE CARE

STAFF INTERVIEW

1. INTRODUCTION & SETTING GROUND RULES (5 MINS)

Thank the participant for his/her availability, introduce researchers and explain:

- Nature and focus of research i.e. the interview is about discussing the participant's experience of delivering the intervention that aims to facilitate patient involvement in decision making about their care.
- Confidentiality: The name of the participant will only be known by the researchers in the present interview and not be revealed to anyone
- The session will be audio-recorded, transcribed and analysed by researchers using NVivo software, one of the most used widely software for qualitative analysis
- The participant will be identified by an ID and all potentially identifying information will be removed

GROUND RULES

- You do not have to share any information that you don't want to
- There are no right or wrong answers
- All conversations must remain confidential
- Mobile phones should be off or on silent-vibrate whilst the group is working
- Any questions?
- Start tape recorder

2. BACKGROUND (5 MINS)

Aim: Icebreaker

- Ask the participant to say a brief word about themselves:
    - First Name
    - Whether they have taken part in an interview before

3. DISCUSSING BENEFITS & PROBLEMS OF IMPLEMENTING THE INTERVENTION (UP TO 30 MINS)

Aim: To understand participants' experiences of delivering the intervention, focusing on the benefits and problems of implementing the intervention.

This intervention is aimed to facilitate patient involvement in the decision making process of the patient's coercive care.

- What do you think are the benefits of implementing this intervention for patients who are involuntarily admitted?
- Did you encounter any problems during the intervention?

  How did you deal with these problems?
  Were there challenges related to the type of patient?
  Were there any problems related to the ward environment or type of ward?
- Was the decision making topic guide (i.e. list of items) helpful in guiding the conversation?
- What do you think were the benefits (and/or problems) of including carers in the intervention session?

4. DISCUSSING PRACTICAL ISSUES RELATED TO THE INTERVENTION (UP TO 40 MINS)

Aim: to understand participants' opinions on the practicalities of delivering the first intervention session.

- How does the intervention fit with the constraints of your day to day in-patient practice?
- Do you think that it would be feasible for this intervention session to be implemented as normal practice as soon as a patient is involuntarily admitted?
- How are the decisions made during the intervention session communicated to other members of staff?
- How did you deal with perceived disagreement with a patient (and/or carer) during the intervention session?

5. CONCLUSION / DEBRIEF (10 MINS)

- Thinking about the conversations we have had today about the plan, which **one thing** do you think is the biggest benefit of the intervention session?
- What do you think is the most important barrier to it?

- Thank participant for his/her contribution
- Remind participant that all information is confidential
- Information on project timescale (analysis of data, preparation of a report)
